# Cognitive Aging Revisited: A Cross-Sectional Analysis of the WAIS-5

**DOI:** 10.3390/jintelligence13070085

**Published:** 2025-07-12

**Authors:** Emily L. Winter, Brittany A. Dale, Sachiko Maharjan, Cynthia R. Lando, Courtney M. Larsen, Troy Courville, Alan S. Kaufman

**Affiliations:** 1 School of Health Sciences, Touro University, 3 Times Square, New York, NY 10036, USA; smaharja5@student.touro.edu (S.M.); clando@student.touro.edu (C.R.L.); 2 Department of Special Education, Ball State University, Muncie, IN 47306, USA; badale@bsu.edu (B.A.D.); cmlarsen2@bsu.edu (C.M.L.); 3 Pearson Assessments, 927 East Sonterra Boulevard, San Antonio, TX 78258, USA; troy.courville@pearson.com; 4 Department of Educational Psychology, University of Connecticut, Storrs, CT 06268, USA; alan.kaufman@uconn.edu

**Keywords:** cognitive aging, WAIS-5, cross-sectional design, verbal abilities, nonverbal abilities

## Abstract

Historical cross-sectional approaches examining cognitive aging consistently reveal a pattern of steady decline on nonverbal problem-solving, speeded tasks, and maintenance on verbal tasks. However, as measures developed and broadened the factor structure to align with Cattell–Horn–Carroll (CHC) theory, and age ranges were extended from 75 to 90 years, a more nuanced approach to cognitive aging emerged. The present study, using the Wechsler Adult Intelligence Scale, Fifth Edition (WAIS-5), examined the cognitive aging process through a cross-sectional approach. WAIS-5 normative sample data (aligned with the 2022 U.S. census) were obtained from the test publisher. The sample included adult participants aged 20–24 through 85–90 (n = 1660), which were mapped into 11 age groups. Using post-stratification weighting to control for educational attainment, cognitive decline was observed throughout aging; verbal skills were maintained longer than other abilities, while processing speed declined steadily and rapidly from young adulthood to old age. Working memory was vulnerable to the aging process but demonstrated slower patterns of decline than the other vulnerable abilities. Fluid reasoning and visual spatial skills (although aligning with separate CHC broad abilities theoretically) were strikingly similar in their pattern of decline across a person’s lifespan. Results are highly consistent with the large body of cross-sectional research conducted during the previous generation by Salthouse and his colleagues, as well as other teams of researchers.

## 1. Introduction

With aging, the human brain changes both in its physical structure and in its cognitive functioning, as evidenced through cognitive skill decline in decision-making, memory, and attention. The term “cognitive aging” defines the gradual and variable changes related to aging, specifically in intellectual abilities. With advances in health, environment, and education, extensive research has shifted the perspective away from the notion that all age-related changes are correlated with an inevitable decline (Committee on the Public Health Dimensions of Cognitive Aging et al. 2015). For instance, Rowe and Kahn (1987) introduced concepts such as “usual” and “successful aging” to emphasize the complexity and variability observed in several dimensions of cognition. In sum, the study of cognitive aging aims to differentiate the changes that align with aging compared to those that may be associated with neurodegenerative disease.

Prior research has demonstrated specific domains of cognitive function consistently affected by aging (Schaie and Hofer 2001). Memory, for instance, has been studied extensively, with working memory frequently showing significant age-related declines, especially for tasks requiring higher executive control, such as recalling a series of digits backward (i.e., Wechsler’s Backward Digit Span; Borella et al. 2020). Processing speed decline has also been extensively explored as related to aging, with older adults performing significantly slower than younger adults on measures of processing speed, even when controlling for the considerable differences in their fine-motor dexterity (Ebaid et al. 2017).

### 1.1. Wechsler’s Adult Scales

The cognitive aging process highlights the fact that cognitive abilities fluctuate throughout a person’s lifespan (Kaufman 2021). For instance, “maintained” abilities, including language concepts and factual knowledge, tend to increase through late middle age, while working memory and the more “performance” abilities, such as fluid reasoning, visual–spatial processing, and processing speed, tend to decline starting at younger ages (Kaufman et al. 2016; Salthouse 2019). This differential pattern of decline for the abilities measured by Wechsler’s adult scales has been demonstrated consistently across the revisions and standardizations of Wechsler’s scales, from the WAIS to WAIS-R to WAIS-III to WAIS-IV (Wechsler 1955, 1981, 1997, 2008). With the newly released fifth edition of the WAIS (Wechsler et al. 2024a, 2024b), the following question remains: do these patterns of aging continue with updates to the measure, especially with a new five-factor structure for the WAIS-5: Verbal Comprehension Index (VCI), Visual Spatial Index (VSI), Fluid Reasoning Index (FRI), Working Memory Index (WMI), and Processing Speed Index (PSI; Wechsler et al. 2024a, 2024b), as well as for data collected post-pandemic?

### 1.2. Evolution from WAIS to WAIS-5

Originally derived from the Wechsler–Belleview Form I (Wechsler 1939), the Wechsler Adult Intelligence Scale (WAIS; Wechsler 1955) was normed on a stratified sample in the United States as opposed to one small geographical area (a novel concept at the time). In the many revisions of the WAIS over the past half-century, the measure has been adapted, updated, and improved with each passing version, growing in its strength, prevalence of use as a neuropsychological tool, and in its profound influence on psychology research and clinical application. Presently, the shift from the WAIS-IV to the WAIS-5 includes the adjustment from 10 to 7 core subtests used to calculate a Full-Scale IQ (FSIQ), with the addition of new supplemental subtests, thus improving its clinical utility and interpretive depth with non-verbal and motor-reduced measures; further, there is broader construct coverage for the processing speed, working memory, and fluid reasoning domains (Wechsler et al. 2024a, 2024b). The WAIS-5 features updated norms and streamlined administration for shorter administration times as well as features data collected exclusively following the COVID-19 pandemic. Most notably, the WAIS-5 includes a shift from a four-factor to a five-factor model. The previous factors were VCI, Perceptual Reasoning (PRI), WMI, and PSI. The WAIS-5 split and restructured the PRI into the VSI and FRI, aligning more closely with CHC broad abilities. Furthermore, other key modifications in the WAIS-5 include an update of the theoretical foundation, an increase in the appropriateness of developmental changes, increased user-friendliness, improved psychometric properties, and enhanced clinical utility (see Wechsler et al. 2024a, 2024b for details).

### 1.3. Information on Wechsler’s Full-Scale IQ

The WAIS-5 measures a person’s ability (a) to problem-solve using language and verbally presented information (VCI); (b) to problem-solve using primarily visually presented information, such as through spatial puzzles, or holding information within the “mind’s eye” (VSI); (c) to problem-solve with novel information (FRI); (d) to hold information, presented visually or orally, within the mind and use it to solve a new problem (WMI); and (e) to rapidly solve problems with known, or easy-to-access, information (PSI). All of these skill areas are summarized as primary index scores that collectively compose a nuanced look into parts of an individual’s global intelligence, as reflected in FSIQ, which provides an overall score of seven of the primary subtests.

FSIQ represents general intellectual ability, which is a key feature in illustrating an individual’s overall cognitive functioning (Wechsler et al. 2024a, 2024b). Previous research has often prioritized analyzing ability at the composite level, suggesting that FSIQ may overgeneralize an individual’s cognitive performance as a composite of starkly different abilities (as discussed in Kaufman et al. 2016; see McGill 2016), although this remains a debated topic among psychologists. An overall numeric value of intelligence, or *g* factor, has been included in intelligence models since their inception. However, there is frequent disagreement among prominent theorists on the use and relevance of measuring a stratified *g*, with some arguing against it (e.g., Horn 1989) and others in defense (Carroll 1997; Jensen 1998). This, in turn, led to the frequent ignoring of *g* in the CHC model and, subsequently, underplaying FSIQ. Meanwhile, Wechsler himself was a proponent of *g* theory (Kaufman et al. 2009), even though his multi-scale tests encouraged profile interpretation, and some researchers have extolled FSIQ’s psychometric excellence and necessity in Wechsler models, usually to the exclusion of profile interpretation (see Canivez and Watkins 2010a, 2010b; Canivez and Kush 2013).

Indeed, the comprehensive nature of FSIQ is often featured in the identification of giftedness and in the monitoring of Flynn effect advances and its impact on intellectual disability court rulings (Flynn 2009; Kaufman et al. 2009; Lichtenberger and Kaufman 2013). More recently there has been an apparent shift in the size of the Flynn effect, leading to questions of alterations to the current system of legal decision-making (Platt et al. 2019; Trudel et al. forthcoming; Winter et al. 2024).

### 1.4. Educational Attainment and Aging Research

Educational attainment, the highest level of education completed, has been linked to higher cognitive function performance later in life, a concept known as the “cognitive reserve hypothesis” (Ackerman 2005; Lenehan et al. 2015; Wilson et al. 2009). However, the extent to which educational attainment is positively associated with cognitive aging remains inconsistent in the literature. Few studies suggest that higher educational attainment delays cognitive decline (Bosma et al. 2003) or postpones its onset (Clouston et al. 2020). In contrast, most studies provide limited support that higher educational attainment significantly slows the rate of cognitive decline over time (Christensen et al. 1997; Der et al. 2010; Tucker-Drob et al. 2009; Van Dijk et al. 2008; Wilson et al. 2009). For instance, meta-analytic findings suggest that while education plays a critical role in maintaining higher levels of cognitive performance, education does not demonstrate a consistent association with the rate of cognitive aging (Seblova et al. 2020). These findings underscore that educational attainment is strongly linked to the level of cognitive functioning throughout one’s lifespan but not necessarily to the trajectory of cognitive decline.

The majority of studies on cognitive aging rely on a combination of tools such as measures of crystalized intelligence (Christensen et al. 1997), Mini-Mental State Examination (Bosma et al. 2003; Christensen et al. 1997; Proust-Lima et al. 2008; Van Dijk et al. 2008; Wilson et al. 2009), or memory subtests from psychological assessments (Bosma et al. 2003; Clouston et al. 2020; Van Dijk et al. 2008; Wilson et al. 2009). However, few studies have utilized comprehensive cognitive assessments to examine the relation between educational attainment and cognitive aging. For instance, Harrsen et al. (2021) assessed Danish participants born in 1914 using the Wechsler Adult Intelligence Scale (WAIS) between ages 50 to 85. Participants were categorized based on their educational attainment (e.g., those with more than seven years of education and a formal school exam and those with seven or fewer years of education without a formal exam). Higher levels of education were associated with a steeper cognitive decline trajectory. Still, despite the steeper decline, participants in the higher educational attainment category had higher mean IQs at all follow-up points. A noted limitation of the study was the failure to account for the Flynn effect in the statistical analysis. Kaufman et al. (2016) found similar findings in their cross-sectional study utilizing three WAIS editions (i.e., WAIS-R, WAIS-III, WAIS-IV). Higher educational attainment correlated with higher Full-Scale IQs (FSIQ). Nonetheless, the pattern of cognitive ability differences across age ranges remained essentially the same for individuals, regardless of their educational background.

For the present study, the most pertinent and pervasive research findings were as follows: (a) educational attainment has been shown, time and again, to correlate significantly and substantially with children’s and adults’ performance on IQ tests; (b) in general, from a societal and historical perspective, at any given point in time, young adults on average have the highest degree of formal education, whereas older adults have the least. As such, education is a powerful cohort effect that must be controlled to the degree possible when comparing the intelligence of adults in different age groups. Without such control, it would be impossible to infer the degree to which cognitive decline is due to age, as opposed to reflecting the lower educational attainment of older individuals. 

### 1.5. Cross-Sectional Design

Aligning with much prior research on aging using the Wechsler scales, the present study applied a cross-sectional design (i.e., between-person; Salthouse 2016), comparing various age groups all measured at one point in time (Kaufman 2001). Cross-sectional approaches have demonstrated consistent findings between aging and cognitive decline (although not without methodological limitations; see Salthouse 2016 for more). Schaie (2012) reported that the cross-sectional approach is helpful when addressing questions regarding studying interindividual differences. Cross-sectional design, however, does not control for cultural factors (i.e., generational factors), such as pivotal experiences in specific generations, including the COVID-19 pandemic. Salthouse, across much of his prolific work, discusses the criticality of methodology to answer the following question: when does age-related decline begin (which Salthouse calls a “monotonic” pattern)? In general, prior cross-sectional research shows increases in crystallized abilities from adolescence through to one’s 50s or 60s, and maintenance until about age 75, before these abilities decline rapidly into old age (Kaufman and Horn 1996); by contrast, fluid abilities and processing speed peak during one’s 20s, and then display a steady and often precipitous decline through to old age (Kaufman et al. 2016; Salthouse 2010, 2014). Importantly, Salthouse (2014) notes that these cross-sectional findings align closely with cognitive declines observed in “quasi-longitudinal” studies of Wechsler’s scales, which follow the changes in ability level of independent samples from the same cohorts (years of birth) over decades (Kaufman 1990, 2001; Lichtenberger and Kaufman 2013). 

### 1.6. Purpose of the Present Study

The current study was conducted to consider cognitive aging from a cross-sectional design standpoint given the recent release of the WAIS-5 post-pandemic (Wechsler et al. 2024a, 2024b). Prior literature examining cognitive aging has supported the application of both cross-sectional and quasi-longitudinal designs (Kaufman 2001; Lichtenberger and Kaufman 2013; Salthouse 2019). Given the changes in the newest edition of the WAIS-5, shifting from a four- to a five-factor model, the present study will provide new insights into the process of aging from a five-factor, cross-sectional approach.

## 2. Materials and Methods

### 2.1. Instruments

The present study utilized the WAIS-5 (Wechsler et al. 2024a, 2024b), a highly used measure of intelligence with strong demonstrated evidence of reliability and validity displayed across all ages (i.e., 16 to 90 years). Index reliability coefficients ranged from 0.90 to 0.98 on the WAIS-IV and 0.90 to 0.97 on the WAIS-5 (see Table 4.1 in Wechsler 2008; Wechsler et al. 2024b). For detailed information on evidence of reliability and validity for this measure, see Chapter 4 of the WAIS-5’s Technical and Interpretative Manual (Wechsler et al. 2024b). For further information concerning updates to the fifth edition, a comprehensive review is available in the manuals (Wechsler et al. 2024a, 2024b).

### 2.2. Participants

The adult portion of the WAIS-5 standardization sample was used for the present study, a diverse nationally representative sample of adults across the 20–90-year lifespan. Specifically, 180 participants comprised each age group for individuals aged 20–24 through 85–90 (*n* = 1660), which mapped 11 different age groups. Data were gathered by NCS Pearson to be representative of the U.S. Census at designated intervals—some at 5-year intervals and some at 10-year intervals. For more information regarding the demographic breakdown of the sample, please see the WAIS-5’s Technical and Interpretative Manual (Wechsler et al. 2024b). 

### 2.3. Procedures

The present cross-sectional analyses were completed using the standardization normative data collected from the WAIS-5 (collected between February 2023 and January 2024; Wechsler et al. 2024b). The current study uses methodology employed by Kaufman and colleagues (Kaufman 2001; Kaufman et al. 1989), who used cross-sectional designs to examine differences in IQ on the WAIS-R and WAIS-III. Data analysis was conducted by the sixth author as part of the standardization process for the WAIS-5 at Pearson Assessments, with approval from the Pearson Clinical Assessment Scientific Council meeting on 15 September 2020. To learn more about the Scientific Council, see here: http://www.pearsonassessments.com/professional-assessments/research/research-engagement-portal/who-we-are.html (accessed on 7 July 2025). The study was not preregistered. Analyses were completed in Excel.

Table 1 presents a shift in proportion of the population with higher education levels. This table is a visual representation for the rationale for post-stratification weighting. Poststratification weighting was used in the present analyses given the higher educational level seen in the sample for the WAIS-5 in comparison to the prior edition of the test (e.g., WAIS-IV). Post-stratification weighting is a statistical process, often employed in survey research, to correct bias when a demographic group is overrepresented or underrepresented in the sample. A general weight formula is w_i_ = P_i_/S_i_, where P_i_ is the population proportion and S_i_ is the sample for a given stratum. For this study, the demographic of interest was age by educational level. To calculate the weights the following process was used: (1) Calculate the proportion of examinees in each educational group for ages 25–34, treat this group as the population proportion. (2) Calculate the proportion of examinees in each educational group for each of the 11 age groups. (3) Divide the population proportion for each educational group by the proportion of examinees in each educational group for each age group. These three steps lead to an educational weight for each examinee based on the examinee’s age group and educational level.

## 3. Results

Mean standard scores for all age groups are based on the conversion tables for ages 25 to 34-year-olds, which provides a common yardstick for comparing the 11 age groups’ gains and declines between young adulthood and old age; these means were provided by Pearson for each of the WAIS-5 composite scores, separated by age group. Educational-adjusted and non-educational-adjusted means for WAIS-5 FSIQ, VCI, VSI, FRI, WMI, and PSI are presented in Table 2 for ages 20–24 through 85–90.

**Figure 1:**
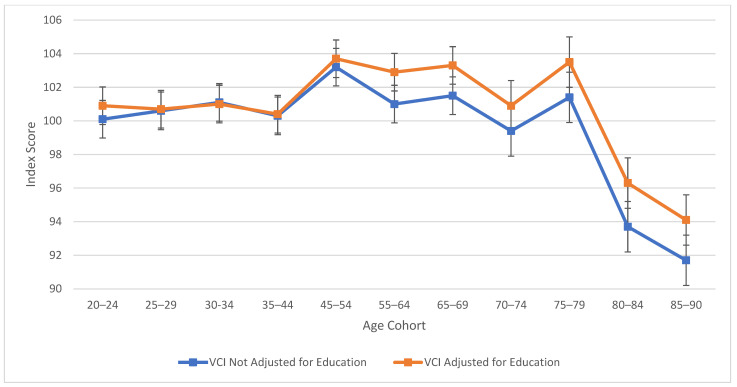
Age differences in verbal comprehension—WAIS-5 VCI by age between 20 and 90 years. Note: Error bars indicate the standard error of the mean.

**Figure 2:**
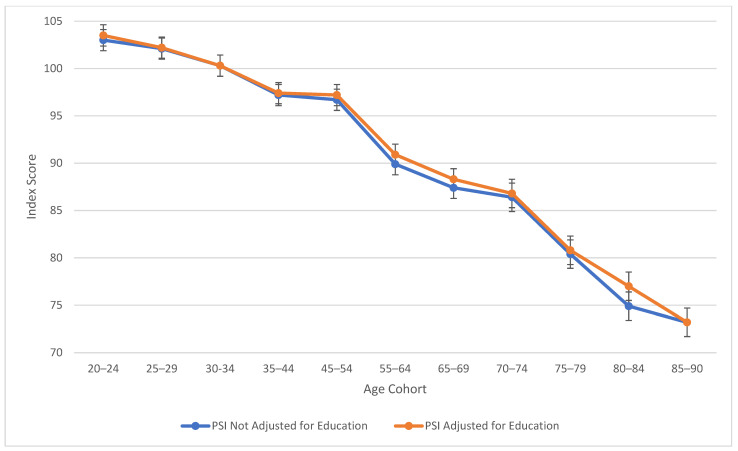
Age differences in processing speed—WAIS-5 PSI by age between 20 and 90 years. Note: Error bars indicate the standard error of the mean.

As observed in Figure 1, verbal comprehension skills peaked at ages 45–54 for both education-adjusted and non-education-adjusted means, remaining relatively stable through ages 70–74. There is a large decline in verbal skills at age 80. These results align with findings from previous cross-sectional studies (Kaufman 2001; Kaufman and Horn 1996) in terms of the “crest” and decline; however, the stability in the profile throughout middle age was relatively novel in comparison to the WAIS-III’s findings (Kaufman 2001), which indicated a more drastic downturn. It is important to note that from the third to the fifth edition of WAIS, Verbal Comprehension (WAIS-5) and Verbal IQ (WAIS-III) are not identical constructs. In contrast to the age differences that characterize VCI, Figure 2 displays the consistent decline in processing speed ability across the age groups, which demonstrates steady decline starting within the youngest age group (the 20–24 age bracket).

**Figure 3:**
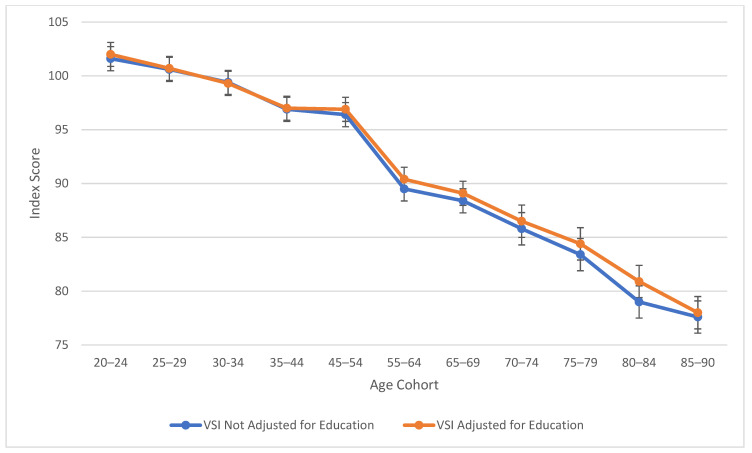
Age differences in visual spatial ability—WAIS-5 VSI by age between 20 and 90 years. Note: Error bars indicate the standard error of the mean.

**Figure 4:**
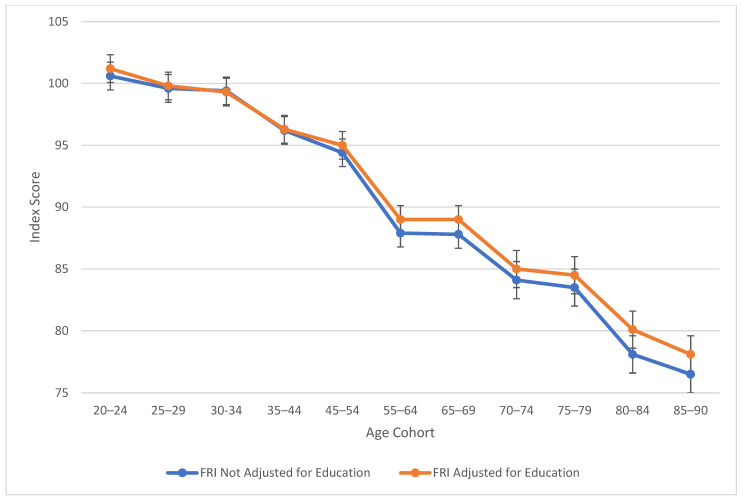
Age differences in fluid reasoning—WAIS-5 FRI by age between 20 and 90 years. Note: Error bars indicate the standard error of the mean.

**Figure 5:**
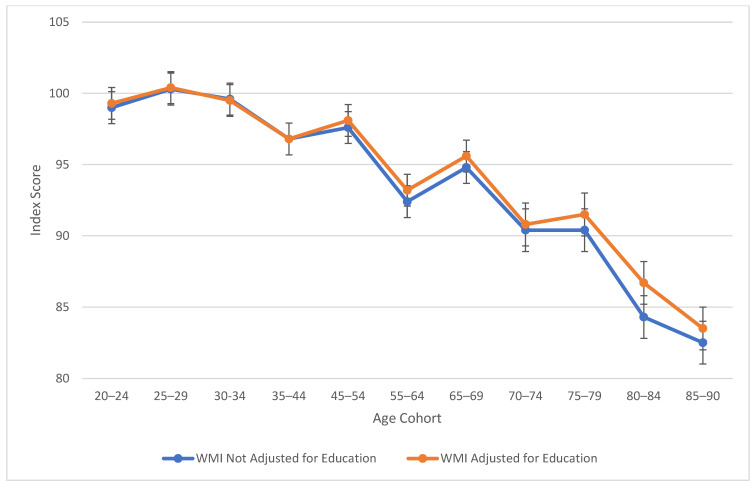
Age differences in working memory—WAIS-5 WMI by age between 20 and 90 years. Note: Error bars indicate the standard error of the mean.

Table 2 also explores similarities between visual spatial, fluid reasoning, and working memory abilities. All three cognitive abilities show a decline in cognitive skills across ages, which is seen more sharply after age 45 (see Figure 3, Figure 4 and Figure 5). At this point in age, there is a larger drop observed in cognitive skills. Working memory maintains the highest score throughout aging; it is a vulnerable ability, but not to the extreme extent of fluid reasoning and visual spatial skills. As visually noted in the charts, VSI and FRI (Figure 4 and Figure 5) have periods of alternating decline and plateaus, whereas WMI displays a “spikier” pattern of decreases, increases, and plateaus. For all figures, note that standard scores for all age groups are based on conversion tables for ages 25–34 to permit comparisons across the wide age range.

Table 3 highlights the effect sizes connected to age-by-age alterations to the WAIS-5 composite scores. Age groups that had the highest mean education-adjusted IQ (see Table 2) were determined as the “Peak Age.” The Peak Age was 45–54 for VCI, 20–24 for VSI, PSI, FRI, and FSIQ, and 25–29 for WMI. The mean education-adjusted IQ noted for each age group was then subtracted from the Peak Age group’s mean, then divided by 15 to convert to units of standard deviation. Effect sizes based on the unit of standard deviation (McLean 1995) have the following classifications: small (<0.50), moderate (0.50–1.00), and large (>1.00).

Outside of verbal comprehension skills (i.e., crystalized intelligence), the “Peak Age” is the youngest group (20–24) for visual spatial, fluid reasoning, processing speed, and Full-Scale IQ, and the second youngest group (25–29) for working memory. Verbal comprehension, on the other hand, had an older “Peak Age” (ages 45–54).

Working memory maintains the longest among the vulnerable abilities, showing the greatest decline after age 70. Visual spatial and fluid reasoning function similarly in their decline. Processing speed shows the largest difference scores from the Peak Age group, with large effect sizes for the standard deviation differences present starting in the mid-60s. For verbal comprehension, only the highest age group showed a moderate difference between “Peak Age” VCI.

## 4. Discussion

Prior scholars using cross-sectional methodology who examined cognitive aging have long proposed the concept of maintained crystallized abilities, at least through late middle age, contrasted with vulnerable working memory, processing speed, and problem solving (visual spatial and fluid); the sometimes dramatic decline in vulnerable abilities has shown to begin early (in one’s 20s) and continue steadily and relentlessly through to the oldest age group tested (Kaufman 2001, 2021; Kaufman et al. 2016; Salthouse 2010, 2014, 2016, 2019). The same findings have been observed in quasi-longitudinal investigations, where independent samples from the same age cohorts have been followed for a dozen or more years (Kaufman 1990, 2001; Lichtenberger and Kaufman 2013; Salthouse 2014). Though a great majority of investigations have been conducted with Wechsler’s individually administered adult scales, the findings generalize to an array of IQ tests, whether comprehensive (e.g., Kaufman and Horn 1996) or brief (e.g., Wang and Kaufman 1993), or administered in group format (e.g., Schaie and Hofer 2001; Kaufman and Horn 1996). Crystallized abilities, so often interpreted as being maintained throughout one’s lifespan—if not increasing, then at least reaching a plateau—were found to decline precipitously after age 75, no different from the so-called vulnerable abilities. 

The results of the present study are consistent with the findings from the wealth of previous cross-sectional literature, including the precipitous plunge of all WAIS-5 Scale Indexes, including VCI, at ages 75 and older (see Figure 1, Figure 2, Figure 3, Figure 4 and Figure 5). Though the findings do not depart from the bulk of the previous cross-sectional investigations, the present study adds to the literature in several ways: (a) the data were obtained on the WAIS-5, which has already assumed the mantle of the most popular clinical test of intelligence for adults in the United States, and will replace the WAIS-IV as the go-to clinical test worldwide as translations and adaptations of the WAIS-5 proliferate; (b) the data were obtained in 2023, and therefore reflect a snapshot of cross-sectional aging patterns in the present; (c) all data were obtained post-COVID-19 pandemic; the replication of pre-pandemic data within the contemporary adult population suggests that whatever effect the pandemic might have on children’s intelligence, it might not be as potent a variable for the adult population (Trudel et al. forthcoming observed changes in the Flynn effect for 16 year olds that suggested a possible negative impact from the pandemic on acquired knowledge and verbal abilities); (d) a more sophisticated control of educational attainment was applied than has been used in previous cross-sectional investigations, namely post-stratification weighting, providing an excellent control of this potent, and potentially contaminating, cohort variable; and (e) FSIQ was also evaluated across the age range, with an educational control, an analysis that is valuable for situations in which FSIQ assumes prominence, such as capital punishment cases; when the differential rates of decline of the five abilities measured by the WAIS-5 are integrated into a single 7-subtest global score, the FSIQ is seen to maintain through age 54 (mean for ages 45–54 = 97.8) before declining steadily to a “borderline” mean of 77.8 at ages 85–90 (see Table 2). 

As seen across Figure 1, Figure 2, Figure 3, Figure 4 and Figure 5, obtained and education-adjusted index scores do not differ drastically, although the impact of education is especially notable for VCI for adults aged 55 to 90 years. Apart from VCI, the fairly small adjustments to obtained standard scores based on differences in educational attainment are consistent with previous literature. It would be interesting to compare cognitive changes across the age range for homogeneous educational attainment samples (e.g., high school dropouts, high school graduates, college graduates). Ample evidence shows that those with more educational attainment start off with higher cognitive scores than those with lower education (e.g., Lichtenberger and Kaufman 2013). However, further data are still needed to support or refute the contention that higher educational attainment slows cognitive decline (Seblova et al. 2020), an idea referred to the “active cognitive reserve” hypothesis. The present samples were not large enough to test this hypothesis. 

Within the context of the VCI results, when comparing VCI to older data that use Verbal IQ, it is important to consider that VIQ and VCI are different constructs. That said, the VCI and VIQ correlate highly with each other (*r* = 0.89; Wechsler 2008); thus, we hypothesize that the maintenance is unrelated to the changes in construct. Rather, the answer potentially lies in the greater educational attainment of older people in 2023 (63% are college educated) versus 1995 (14% are college educated; Wechsler 1997). Verbal knowledge and verbal problem solving are intimately related to educational attainment. The greater educational attainment of people in late middle age and older age in contemporary 2020 society perhaps helps with the maintenance of crystallized abilities longer than in previous generations. Even a sophisticated control for educational attainment would not alter that fact. Furthermore, the control for educational attainment has less impact when each age group through to the 70s has the same level of education.

In sum, considering the findings in context of the wealth of prior research, the fact remains that the present results are consistent with previous cross-sectional results—from every Wechsler scale since Wechsler–Bellevue, along with the results of Salthouse’s extensive analysis of a different individually administered battery of separate abilities (2010, 2014, 2016, 2019); Woodcock–Johnson results (McArdle et al. 2002); and adult versions of Kaufmans’ tests (Kaufman and Horn 1996; Kaufman et al. 1996, 2008; Wang and Kaufman 1993)—providing strong evidence that intelligence is indeed measured by Wechsler’s scales. Furthermore, these data highlight that the core definition of IQ does not change from (a) Wechsler scale to Wechsler scale, (b) Wechsler scale to Woodcock or Kaufman scale, (c) clinical test of intelligence to laboratory developed test of intelligence (i.e., Salthouse and colleagues at University of Virginia), or (d) from the 1930s to the 1950s, the 1980s, the 1990s, the 2000s, the 2010s, or the present.

Regarding evidence of validity, three of the crucial aspects of construct validity for an IQ test, according to Susanna Urbina (2014) and her mentor—legend Anne Anastasi—are (a) the internal consistency of its components, (b) factor analysis that ties each factor to a theoretical construct, and (c) lawful age changes across the age range. Point “a” is evident from moderate but substantial correlations across subtests and indexes and from the emergence of strong *g* factors in all exploratory and confirmatory factor analyses. Point “b” is evident from the results of EFA and CFA, as well as the ability represented by each of the five separate WAIS-5 Indexes corresponds to a major broad ability in CHC theory and the working memory and processing speed factors aligning well with the relevant theories of working memory and neuroscience on which the test battery is partially built upon. Further evidence of the construct validity noted by Urbina is an IQ test’s ability to distinguish among groups who are known to reach IQs that are higher or lower than those of typically developing adults. This evidence is provided by the data on clinical groups such as individuals with Intellectual Developmental Disorders, giftedness, and brain injuries, as examples. Furthermore, point “c” is one of the main purposes of this study—to provide evidence of validity for the WAIS-5 Indexes by demonstrating age changes that are relevant in view of theory, findings from neurology, and 80+ years of high-level research.

Practically, these findings highlight that, on average, older adults, especially those in their late 70s and 80s, demonstrate poor functioning in all aspects of widely used IQ tests—in this case, the popular WAIS-5. As more leaders continue to work into older age (well into their 80s)—such as federal judges and elected officials—these findings underscore Kaufman’s (2021) challenging of the present guidelines for lifetime appointments and the potential need for the routine cognitive assessment of our country’s greatest leaders to ensure that they possess the required mental fitness for the high-stress, high-impact work they carry out. In practical everyday life, this research highlights the potential clinical utility of cognitive assessment for use in mental fitness testing for involvement in tasks that require strong cognitive skills, such as having a driver’s license.

### 4.1. Limitations

Although there are many strengths in the cross-sectional design, an immediate limitation is that the design compares adults from different age cohorts, and any observed differences might be contaminated by cohort difference owing to societal or generational changes or events (e.g., war, pandemics, medical advancements, Schaie 2012). We controlled for one known, and powerful, cohort variable, namely educational attainment; however, cohort variables as a rule are not so easily quantifiable and are often unknown. Furthermore, we opted to eliminate the two youngest age groups (16–17; 18–19), thereby truncating the age range of our sample to permit the application of the educational attainment control (ages 16–19 were stratified by parent’s education).

Finally, the normative sample collected by NCS Pearson had exclusionary criteria, such as individuals with intellectual disabilities, psychiatric disorders, and neurological conditions. By omitting these individuals’ data, the picture painted does not include groups of individuals who may experience different patterns of cognitive decline with aging than more neurotypical adults.

### 4.2. Future Directions

Future research may consider addressing some of the limitations of the cross-sectional methodology by conducting longitudinal or quasi-longitudinal research designs (Kaufman 2001; Salthouse 2014; Schaie 2012). Expanding the research to other instruments for assessing adult intelligence, most notably the new Woodcock–Johnson V (McGrew et al. 2025), would be extremely valuable, especially since it includes co-normed measures of academic achievement and is based on the CHC theoretical model of intelligence. Furthermore, exploration of gender differences within aging within cross-sectional and quasi-longitudinal approaches would be interesting for future research. Additionally, prior research has included factors such as social connection in the cognitive aging process, which future research examining cross-sectional aging patterns may wish to more explicitly include as a variable (Sörman et al. 2017). Future researchers working on collecting normative sample data should consider gathering information on mild cognitive impairment and pre-clinical dementia (as well as other vascular risk factors—for risk of vascular dementia [body mass index, metabolic profile; Tariq and Barber 2018]—and main bio indices such as APOE e4) to aid in determining exactly who should and who should not be included in a representative sample of typical adults. Finally, future research may discuss the comparison between epidemiological studies to consider risk factors’ role in the cognitive decline of specific cognitive arenas (Baumgart et al. 2015). Scholars may also be interested in comparing the neurodegeneration decline profile to subjective cognitive decline and mild cognitive impairment due to preclinical Alzheimer’s disease in order to better understand if the same pattern of decline is observed in each cognitive ability area (Cloutier et al. 2015). These data would be interesting to understand and compare in the neuropathological spectrum, from patterns of aging observed in the preclinical to disease stages.

## 5. Conclusions

The present study, using cross-sectional methodology, conforms with the results of a huge body of prior research (Kaufman 2021; Salthouse 2019), extending these findings to data obtained in 2023, post-pandemic, on the latest version of Wechsler’s adult scales, the WAIS-5. Verbal skills were maintained through late middle age, but joined the vulnerable abilities of working memory, fluid reasoning, visual spatial ability, and processing speed with its dramatic decline between ages 75 and 90. Although PRI on the WAIS-IV was split into VSI and FRI on the WAIS-5, it is notable that the two new scales, though measuring separate and distinct CHC abilities, displayed virtually identical aging patterns in our analyses. 

## Figures and Tables

**Table 1:** Percentages of adults with different amounts of education.

	0–11		12		1+ Years of College	
Age	2007	2023	2007	2023	2007	2023
20–24	13.0	11.9	30.5	22.8	56.5	65.7
25–29	12.5	7.0	29.0	25.6	58.5	67.9
30–34	12.5	7.4	27.5	22.3	60.0	70.7
35–44	11.5	8.6	30.5	22.3	58.0	69.4
45–54	11.0	10.2	32.0	22.8	57.0	67.2
55–64	13.0	10.2	32.0	27.8	55.0	62.2
65–69	20.0	9.7	34.5	28.9	45.5	61.6
70–74	22.0	10.0	39.0	27.0	39.0	63.0
75–79	28.0	11.0	36.0	29.0	36.0	60.0
80–84	28.0	15.0	36.0	33.0	36.0	52.0
85–90	36.0	16.0	33.0	36.0	31.0	48.0

Note: Percentages may exceed 100% given what was reported in the manual.

**Table 2:** WAIS-5 mean scores for adults aged 20–90—both with and without an adjustment for educational attainment.

Age Group	20–24	25–29	30–34	35–44	45–54	55–64	65–69	70–74	75–79	80–84	85–90
FSIQ	101.1	100.7	100	97.3	97.8	91.5	91.2	88.2	86.8	80.1	77.8
FSIQ Ed-Adjusted	101.8	100.9	99.9	97.5	98.5	92.9	92.7	89.2	88.2	82.5	79.3
FSIQ Difference	0.7	0.2	−0.1	0.2	0.7	1.4	1.5	1.0	1.4	2.4	1.5
VCI	100.1	100.6	101.1	100.3	103.2	101	101.5	99.4	101.4	93.7	91.7
VCI Ed-Adjusted	100.9	100.7	101	100.4	103.7	102.9	103.3	100.9	103.5	96.3	94.1
VCI Difference	0.8	0.1	−0.1	0.1	0.5	1.9	1.8	1.5	2.1	2.6	2.4
PSI	103	102.1	100.3	97.2	96.7	89.9	87.4	86.4	80.4	74.9	73.2
PSI Ed-Adjusted	103.5	102.2	100.3	97.4	97.2	90.9	88.3	86.8	80.8	77	73.2
PSI Difference	0.5	0.1	0.0	0.2	0.5	1.0	0.9	0.4	0.4	2.1	0.0
VSI	101.6	100.6	99.4	96.9	96.4	89.5	88.4	85.8	83.4	79	77.6
VSI Ed-Adjusted	102	100.7	99.3	97	96.9	90.4	89.1	86.5	84.4	80.9	78
VSI Difference	0.4	0.1	−0.1	0.1	0.5	0.9	0.7	0.7	1.0	1.9	0.4
FRI	100.6	99.6	99.4	96.2	94.4	87.9	87.8	84.1	83.5	78.1	76.5
FRI Ed-Adjusted	101.2	99.8	99.3	96.3	95	89	89	85	84.5	80.1	78.1
FRI Difference	0.6	0.2	−0.1	0.1	0.6	1.1	1.2	0.9	1.0	2.0	1.6
WMI	99	100.3	99.6	96.8	97.6	92.4	94.8	90.4	90.4	84.3	82.5
WMI Ed-Adjusted	99.3	100.4	99.5	96.8	98.1	93.2	95.6	90.8	91.5	86.7	83.5
WMI Difference	0.3	0.1	−0.1	0.0	0.5	0.8	0.8	0.4	1.1	2.4	1.0

Note: Standard scores for all age groups are based on conversion tables for ages 25–34 to permit comparisons across the wide age range. Standard deviations for Full-Scale IQ and Index scores are all approximately 15.

**Table 3:** Using post-stratification weighting.

Age Group	VCI Diff	VCI ES	VSI Diff	VSI ES	FRI Diff	FRI ES	WMI Diff	WMI ES	PSI Diff	PSI ES	FSIQ Diff	FSIQ ES
20–24	2.8	0.19	Peak Age		Peak Age		1.1	0.07	Peak Age		Peak Age	
25–29	3.0	0.20	1.3	0.09	1.4	0.09	Peak Age		1.3	0.09	0.9	0.06
30–34	2.7	0.18	2.7	0.18	1.9	0.13	0.9	0.06	3.2	0.21	1.9	0.13
35–44	3.3	0.22	5	0.33	4.9	0.33	3.6	0.24	6.1	0.41	4.3	0.29
45–54	Peak Age		5.1	0.34	6.2	0.41	2.3	0.15	6.3	0.42	3.3	0.22
55–64	0.8	0.05	11.6	0.77 ^a^	12.2	0.81 ^a^	7.2	0.48	12.6	0.84 ^a^	8.9	0.59 ^a^
65–69	0.4	0.03	12.9	0.86 ^a^	12.2	0.81 ^a^	4.8	0.32	15.2	1.01 ^b^	9.1	0.61 ^a^
70–74	2.8	0.19	15.5	1.03 ^b^	16.2	1.08 ^b^	9.6	0.64 ^a^	16.7	1.11 ^b^	12.6	0.84 ^a^
75–79	0.2	0.01	17.6	1.17 ^b^	16.7	1.11 ^b^	8.9	0.59 ^a^	22.7	1.51 ^b^	13.6	0.91 ^a^
80–84	7.4	0.49	21.1	1.41 ^b^	21.1	1.41 ^b^	13.7	0.91 ^a^	26.5	1.77 ^b^	19.3	1.29 ^b^
85–90	9.6	0.64 ^a^	24	1.60 ^b^	23.1	1.54 ^b^	16.9	1.13 ^b^	30.3	2.02 ^b^	22.5	1.50 ^b^

Note: ES = effect size in units of standard deviation; Diff = the difference between mean education-adjusted IQ within the age group who scored the highest (known as “Peak Age”) and the mean education-adjusted IQ earned within each age group. ^a^ = moderate effect size; ^b^ = large effect size.

## Data Availability

Standardization data were from the Wechsler Adult Intelligence Scales, Fifth Edition (WAIS-5) Copyright © 2024. NCS Pearson, Inc. Data are used with permission. All rights are reserved. Data cannot be shared with others outside of the research team. Researchers can request the Pearson Assessments standardization data licenses online at https://www.pearsonassessments.com/forms/standardization-data-license-requests.html (accessed on 7 July 2025).
